# Unravelling Selection Shifts Among Foot-and-Mouth Disease Virus (FMDV) Serotypes

**Published:** 2007-02-11

**Authors:** Damien C. Tully, Mario A. Fares

**Affiliations:** 1 Molecular Evolution and Bioinformatics Laboratory, Biology Department, National University of Ireland, Maynooth, Co. Kildare, Ireland

**Keywords:** FMDV, Adaptive Evolution, Selection shifts, Coevolution

## Abstract

FMDV virus has been increasingly recognised as the most economically severe animal virus with a remarkable degree of antigenic diversity. Using an integrative evolutionary and computational approach we have compelling evidence for heterogeneity in the selection forces shaping the evolution of the seven different FMDV serotypes. Our results show that positive Darwinian selection has governed the evolution of the major antigenic regions of serotypes A, Asia1, O, SAT1 and SAT2, but not C or SAT3. Co-evolution between sites from antigenic regions under positive selection pinpoints their functional communication to generate immune-escape mutants while maintaining their ability to recognise the host-cell receptors. Neural network and functional divergence analyses strongly point to selection shifts between the different serotypes. Our results suggest that, unlike African FMDV serotypes, serotypes with wide geographical distribution have accumulated compensatory mutations as a strategy to ameliorate the effect of slightly deleterious mutations fixed by genetic drift. This strategy may have provided the virus by a flexibility to generate immune-escape mutants and yet recognise host-cell receptors. African serotypes presented no evidence for compensatory mutations. Our results support heterogeneous selective constraints affecting the different serotypes. This points to the possible accelerated rates of evolution diverging serotypes sharing geographical locations as to ameliorate the competition for the host.

## Introduction

Foot-and-Mouth Disease Virus (FMDV), a single-stranded RNA virus, belongs to the Picornaviridae family (Racaniello, 2001). It has a genome of 8.5 kb that is translated into a polyprotein and processed to yield the structural (P1) and non-structural (P2 and P3) proteins (Belsham, 1993). P1 produces the four different capsid proteins (VP1 to VP4). VP1 is the most surface-exposed capsid protein (Acharya et al. 1989) and contains the major antigenic determinants A, C and D (Mateu, 1995). Site D is also formed by residues from VP2 and VP3 and it involves residues of the C-terminus of VP1 (Lea et al. 1994). The antigenic diversity of VP1 makes it an ideal candidate for epidemiological studies (Bastos et al. 2000; Bastos et al. 2001; Sangare et al. 2001; Bastos et al. 2003; Knowles and Samuel, 2003; Sangare et al. 2003).

FMDV is classified into seven immunologically diverse serotypes distributed throughout the world (South Africa Territories (SAT) 1–3, Asia-1, A, O and C). It has an enormous wide spectrum of infection reflecting its genetic and antigenic diversity. O is the most predominant of the serotypes. Type A possesses greater geographical spread than any of the other serotypes. It is also the most antigenically diverse of the Eurasian serotypes with a large amount of variants in Asia, Africa and South America (Ansell et al. 1994; Konig et al. 2001; Araujo, Montassier and Pinto 2002; Tosh, Hemadri and Sanyal 2002). To date according to the OIE/FAO World Reference Laboratory for FMD, the last type C outbreak to emerge was in East Africa and Asia in 1996. The virus type Asia1 is endemic to southern Asia and is now considered the least diverse type with a single subtype (Ansell et al. 1994). South African territories FMDV types (SAT 1–3) have a natural reservoir in the African buffalo (*Syncerus caffer)* (Condy et al. 1985). They are also characterised by their large degree of antigenic diversity (Bastos et al. 2003; Sangare et al. 2003).

A number of evolutionary and population parameters are believed to be responsible for the emergence and dispersal of the different virus serotypes. These constraints can be broadly classed as genetic, for example the ability of the virus to avoid the host immune response, or ecological such as host mobility and population density. Elucidating the mechanisms utilised by viruses that subsequently drive the evolution and infectivity would critically aid in our understanding of viral epidemiology and disease prevention. Recently, Carrillo et al. (Carrillo et al. 2005) have performed an exhaustive comparative genomic analysis to identify novel functional constraints acting on the genome. Conversely, we explored all the available sequences for a gene belonging to the different FMDV serotypes. Analysis of the constraints governing FMDV VP1 protein has been previously conducted to determine the main causes for the emergence of FMDV strain CS (serotype C) (Elena, Gonzalez-Candelas and Moya, 1992). This insightful work yield precise information about the evolutionary parameters governing FMDV serotypes evolution. However, heterogeneity in the evolutionary constraints has not been explored with much detail due to a limitation in the number of sequences and computational tools. Also, no detailed study has been performed before to exhaustively explore other selective constraints at the molecular level in FMDV using an unbiased sample of sequences.

It has been long recognised that the VP1 gene differs in about 30–50% between serotypes (Knowles and Samuel, 2003). We postulate that there are differences in the serotypes ability to infect and spread that may be accounted for by heterogeneity of evolutionary pressures. In this study we test for changes in the selection constraints to which amino acid sites are subjected by examining variations in the evolutionary rates of amino acid sites (selection shifts or site-rate changes between lineages) between the different FMDV serotypes. For this analysis we use the capsid protein VP1, which is essential in cell receptor recognition and escape from the host immune response (Sobrino et al. 2001). We ask the question of what selective constraints have very likely been responsible for the emergence and spread of the different FMDV serotypes. To test for heterogeneity in selective constraints among FMDV serotypes, we conducted several types of analyses. We first determined whether different selective constraints have shaped the evolution of FMDV serotypes by testing for accelerated fixation rates of amino acid substitutions at particular lineages. As a second approach to the identification of selection shifts between serotypes, we also tested functional divergence (within-site amino acid substitution rates changes throughout the phylogeny) between serotypes. Thirdly, amino acid sites responsible for within-serotype accelerated rates of evolution or functional divergence are identified and their biological importance for the ability of the virus to spread and infect examined. Finally, we identified the co-evolutionary relationships between amino acid sites under accelerated rates of evolution or functional divergence and other protein regions as to understand the molecular consequences of such changes in the VP1 protein of FMDV and to speculate about possible epidemiological scenarios responsible for such selective shifts. We perform an exhaustive analysis of how fixation of amino acid replacements by positive Darwinian selection, coevolution between functionally/structurally important residues and functional divergence in VP1 has performed an efficient strategy for FMDV to spread. This study complements previous reports on the evolution and emergence of foot-and-mouth disease serotypes and pinpoints the need for further experimental work on apparently non-important protein regions that extend beyond well-recognised antigenic sites.

## Materials and Methods

### Sequence data

A dataset comprising 665 FMDV VP1 sequences were downloaded from Genebank. This includes all serotypes and only comprises sequences with length greater than 400 nucleotides to account for most of the antigenic regions within the VP1 protein. Table 1 of Supplementary Information describes the serotypes and number of sequences used including the range of sampling times. Accession numbers of the sequences used in this study are provided in Table 2 of Supplementary Information. All serotypes are significantly represented, both geographically and sample wise.

### Sequence alignments and phylogenetic tree inference

Alignments were obtained for VP1 amino acid sequences for each individual serotype using the T-coffee program (Notredame, Higgins and Heringa 2000). We then aligned protein-coding nucleotide sequences for each one of the serotypes by concatenating triplets of nucleotides based on the amino acid alignment. An alignment including the 665 sequences was also built as explained above to test for functional divergence type I (e.g. within-site rates changes among serotypes, see below for a more detailed definition).

To infer an accurate phylogenetic tree, we first estimated the shape parameter (*α*) of the gamma distribution for the amino acid sequence alignment using the program AAml from the PAML package version 3.14 (Yang et al. 2000). This parameter takes into account both multiple amino acid substitutions per site and unequal substitution rates among sites. The substitution model WAG, the model that significantly improves the log-likelihood value of the data after comparison with other models available in the program ModelTest (Posada and Crandall, 1998) using the Likelihood Ratio test (LRT), was used to estimate *α* Finally, we inferred the phylogeny by neighbour-joining (Saitou and Nei, 1987) using gamma-corrected amino acid distances, estimated by maximum likelihood under the WAG model for the dataset of FMDV sequences, using the program MEGA v3.0 (Kumar, Tamura and Nei, 2004). To estimate the support for internal nodes, we inferred the phylogeny using gamma-corrected amino acid distances in MEGA and estimated support values by bootstrap analyses based on 1000 pseudo-replicates. The phylogenetic analyses using gamma-corrected amino acid distances and neighbour-joining yielded the same phylogenetic topology as that based on WAG distances.

### Testing changes in selective constraints using neural networks

To test the biological (functional/structural) importance of particular amino acid sites in the VP1 protein we used two approaches. The first approach was based on the collection of the functional information available for this protein. However, most of the amino acid sites in the molecule have not been tested for their functional importance since studies have been biased towards testing antigenic peptide regions. We hence conducted a neural-network analysis implemented in the program CONSEQ (Berezin et al. 2004). We used as input alignment each one of the FMDV subtype alignments (including each one a minimum of 30 sequences and a maximum of 203 sequences (Table 1 of supplementary information). Briefly, CONSEQ derives automatically a phylogenetic tree from the query multiple sequence alignment and calculates the substitution rates at each position in the sequence alignment by maximum likelihood. A neural network analyses is thereafter conducted by CONSEQ to predict schemes to discriminate between buried and exposed residues. Although the sensitivity regarding the buried and exposed states has been reported to be of about 56% (Berezin et al. 2004), we tested the performance of the test by comparing the predictions to the positions of the amino acid sites in the available crystal structure for the VP1 protein of FMDV. The functional or structural importance of sites was determined by the conservation of the site, with significantly exposed conserved sites being functionally important and conserved buried sites structurally important.

### Characterising the main selective constraints in FMDV serotypes

To test if the different FMDV serotypes have been subjected to different evolutionary constraints at the molecular level, we conducted a precise analysis of selective constraints for each serotype. Normally, the intensity of selection on protein-coding sequences is measured by comparing the number of substitutions per synonymous site (*d_S_*) to the number of replacements per non-synonymous site (*d_N_*) (Kimura, 1977; Sharp, 1997; Akashi, 1999; Crandall et al. 1999; Yang and Bielawski, 2000). The ratio between the two rates( $\omega =\frac{d_{N}}{d_{S}}$) helps to elucidate if the gene has been fixing amino acid replacements neutrally (*ω* = 1), replacements have been removed by purifying selection (*ω* < 1), or mutations have been fixed by adaptive evolution (*ω* > 1). Furthermore, since *d_S_* values are neutrally fixed (except when constraints operate at the RNA level in these sites or when synonymous sites are saturated) they are proportional to the time since the sequences compared have diverged. Consequently, the normalisation of *d_N_* values by *d_S_* makes *ω* independent of time. It has been shown, however, that *ω* is a poor indicator of the action of adaptive evolution (Sharp, 1997; Crandall et al. 1999). The rationale behind this is that punctual events of positive selection of advantageous non-synonymous mutations throughout the evolution of a particular sequence (episodic positive selection) may be hidden by an overwhelming number of deleterious mutations removed by purifying selection from the same protein regions. Consequently, the number of amino acid substitutions will be below the expectation under neutrality despite these punctual events of adaptive evolution and the mean *ω* values will be < 1. A way to tackle this problem is by estimating *ω* value for specific branches of the tree and for each codon region of a protein. Unless branches are pre-specified, using maximum likelihood for this kind of analysis is computationally prohibitive specially when the number of sequences is large and the model is complex.

We have used Kimura-based models to measure the intensity of selection between and within serotypes using the set of 665 sequences. To detect selective constraints we used a sliding-window based approach (Fares et al. 2002) implemented in the program SWAPSC version 1.0 (Fares, 2004). Briefly, the program slides a statistically optimum window size along the sequence alignment to detect selective constraints and estimates the probability of *d_N_* and *d_S_* using a number of simulated data sets. We used 1000 simulated data sets in our analysis obtained using the program EVOLVER from the PAML (Yang, 2000) package, taking as initial parameters the average *ω* transition-to-transversion rates ratio and codon frequency table generated under the Goldman and Yang model (Goldman and Yang, 1994). These parameters were estimated from the real sequence alignment. The program then slides the window along the real sequence alignment and estimates *d_N_* and *d_S_* by the method of Li (Li, 1993). The significance of these estimates is tested assuming a Poisson distribution of nucleotide substitutions along the alignment. This program was also used due to its ability to identify adaptive evolution (*ω* > 1) but also accelerated rates of amino acid substitutions without the restriction of *ω* > 1 (that can be due to either adaptive Darwinian evolution or to the fixation of slightly deleterious mutations by genetic drift), saturations of synonymous sites (where *d_S_* values are underestimated) and hot spots (where both *d_S_* and *d_N_* are significantly high but where *ω* < 1).

### Testing Functional divergence between serotypes

Normally, in phylogenetic tree inference and detection of selective constraints, fast evolving sites and slow evolving sites are considered to remain fast and slow sites, respectively, throughout the entire evolutionary history (for example, the functional importance of sites has not changed along the evolution of the gene, and thus the rate of evolution of that site remained constant), something called the homogeneous gamma model or rates-across-site (RAS) model (Gaucher et al. 2002). If the functional/structural importance of particular amino acid sites changes between lineages, their rates of evolution also changes and a non-homogenous gamma model will explain better the process of evolution.

A non-homogeneous gamma model of evolution can be statistically described by the functional divergence type I (Gu, 1999; Gu, 2001; Wang and Gu, 2001). Because of the significant serotypic and geographic split between FMDV types, we tested whether this split between serotypes was reinforced by serotype-specific amino acid replacement rates. We tested this selection shifts by comparing the likelihood value of the hypothesis of functional divergence to that of a RAS model. This comparison was performed using the Likelihood-ratio test (LRT). We tested functional divergence type I between serotypes using the program DIVERGE version 1.04 (Gu, 1999), respectively. DIVERGE tests for functional divergence type I by estimating the parameter of functional divergence (*θ*), which is equivalent to the anti-correlation in the evolution of the two group of sequences (e.g. FMDV serotypes) being compared. It estimates the posterior Bayesian probability for an amino acid site to belong to the group of sites responsible for functional divergence. These sites share the property of being highly constant in one of the sequence groups and variable in the other, supporting the gain/loss of a functional/structural constraint in one of the groups.

### Identifying coevolution between structural/functional regions in VP1

Amongst FMDV antigenic sites in VP1 protein, Site A is involved in conferring the virus its ability to escape from the immune pressure and to recognise host cell receptors (Aggarwal and Barnett, 2002). Important structural/functional residues spatially adjacent to these sites may have contributed to the antigenicity of the VP1 protein by their functional or structural interaction. In fact, coevolution between sites surrounding functionally important protein regions allows maintaining the structural and spatial stability of active sites (Gloor et al. 2005). Fixation of amino acid replacements in regions with strong functional/structural constraints can improve the biological fitness of the virus (for example to escape from the immune response of the host). Alternatively, slightly deleterious mutations (mutations compromising the structural stability or functional performance of the protein) can be fixed under genetic drift in or nearby functional/structural regions and be compensated by conditionally advantageous mutations (for example, mutations that are advantageous due to their compensatory effect) at interacting sites. The mutational dynamic of the protein (for example, fixation of slightly deleterious mutations, advantageous mutations or compensatory mutations) yields information hence on the evolutionary pathways that a group of sequences (serotype) have undergone and provide support for the functional and/or structural importance of a specific molecular region. Analysis of the different types of mutations also provides an opportunity to test the heterogeneity in the mutational dynamics between serotypes.

We tested intra-molecular coevolution in each one of the serotypes by applying a method based on the information theoretic quantity called mutual information to measure the dependence of mutations in VP1 (see Korber et al. 1993 for details). This method measures the covariation between two amino acid sites using the Mutual Information Criterion (MIC). Mutual information is represented by the entropies that involves the joint probability distribution, P(s_i_, s′_j_), of occurrence of symbol i at position s and j at position s′. The MIC values generated range between 0, indicating independent evolution, and a positive value whose magnitude depends on the amount of covariation. We only included in the analysis those variable positions in the alignment that were parsimony-informative (i.e. they contain at least two types of amino acids and at least two of them occur with a minimum frequency of two). The statistical power of the test is dependent on the number of sequences as well as the amount of variability at each amino acid site. In this case, both parameters were secured since the number of sequences was high and the variability as well. The significance of the MIC values was assessed by randomisation of pairs of sites in the alignment, calculation of their MIC values and comparison of the real MIC values with the randomly generated distribution of MIC values. One million permutations were conducted to test MIC value significance.

## Results

### Heterogeneous patterns of adaptive evolution between serotypes

All serotypes (SAT 1, SAT 2, Asia1, A and O), except C and SAT3, presented evidence of positive Darwinian selection in the VP1 protein (Table 1). We have also analysed the functional/structural importance of these sites using a neural-network based method implemented in the program CONSEQ (Berezin et al. 2004). The assumption made in this program is that conserved regions at the amino acid level may have a functional or structural role. We did not consider the buried/exposed state for the amino acid sites inferred by CONSEQ due to the low sensitivity (56%) reported for this method regarding these states. This analysis supported that regions detected as having undergone adaptive evolution present evidence of a functional or structural role (Table 1).

The comparison of the five serotypes under positive selection highlights diverse patterns of selective constraints at the molecular and phylogenetic levels. Interestingly, all those serotypes under positive selection comprise a similar region around N131- R149 that is located in the immunodominant site A within the G-H loop. However only serotypes A and O, the wider geographically spread FMDV serotypes, have the full RGD motif under positive selection. Moreover, serotypes SAT1 and 2 present evidence of adaptive evolution at overlapping VP1 protein regions. In fact, all of the regions detected in SAT1 to be under adaptive evolution were also found in SAT2 to be positively selected (Table 1). Interestingly, SAT2 presented greater percentage of adaptive evolution compared to SAT1, which correlates with a wider geographical distribution of this serotype in Africa. Conversely, regions under positive selection in the widely geographically distributed serotypes (serotypes A, Asia 1 and O) presented poor overlap. Interestingly, there is more overlap in the regions under adaptive evolution between SAT serotypes and serotypes Asia1, O and A than within widely spread serotypes (for example between Asia1 and O).

If we were to classify these serotypes in terms of percentage of codon sites under positive selection the following is observed: SAT2 > ASIA 1 > A > SAT 1 > O. The difference from SAT 2 to type O represents a 10-fold increase in the percentage of sites under adaptive evolution.

### Evidence for changes in selection constraints among FMDV serotypes

For each individual serotype we applied the neural-network analysis to detect functional/structural sites implemented in the program CONSEQ. This analysis also enabled us to know whether sites under adaptive evolution had any functional/structural role. In the seven serotypes, the RGD motif from the VP1 protein was identified as functionally important, except in the case of Asia1 and A serotypes where only the G144 and D145 were identified as important.

**Figure 1:**
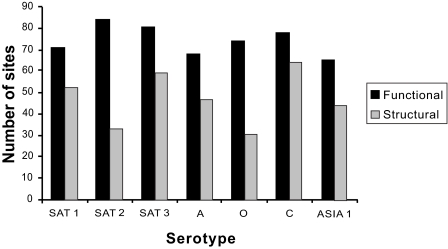
Number of functional (black bars) and structural (grey bars) important sites in each serotype as predicted by the neural-network analysis implemented in the program CONSEQ.

The level of functional important sites varied significantly between serotypes (Figure 1). For example, the variability of the proportion of functionally important sites when comparing the different serotypes was significant (*χ*_6_^2^ = 14.034, *P* = 0.029). Similarly, the proportion of structurally important sites was also significantly different among serotypes (*χ*_6_^2^ = 20.948, *P* < 0.01). Comparison of the number of sites under functional and structural constraints between serotypes uncovers an interesting pattern that suggests certain correlation between the selective constraints acting on VP1 and the functional/structural selection shifts among serotypes. In fact, those serotypes that have not undergone adaptive evolution present the greatest number of functionally and structurally important regions (140 and 145 sites for SAT3 and C serotypes, respectively). The percentage of sites under functional constraints as detected by CONSEQ were not an artefact of the amino acid variability produced by adaptive evolution since all positively selected regions were detected to be functionally/structurally important. Thus, high variability due to positive selection was not leading to an underestimation of functional/structural important sites.

### Functional divergence between FMDV serotypes

FMDV serotypes were compared to each other and regions under functional divergence type I determined. Only amino acid sites showing posterior Bayesian probabilities greater than 0.95 were considered as having been subjected to functional divergence. In all the pairwise comparisons between serotypes, most of the sites involved in functional divergence were found in the carboxy-terminal half of the molecule, affecting antigenic sites A, C and D.

**Figure 2:**
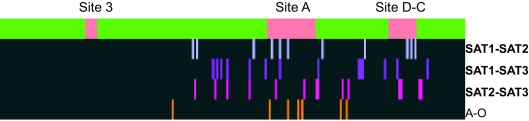
Analysis of functional divergence between FMDV serotypes. The green horizontal bar represents the sequence of the VP1 protein of FMDV, with antigenic sites pink-labelled. Vertical bars in each horizontal lane indicate the approximate location of sites under functional divergence in the serotypes compared. The different comparisons are colour-coded for clarity.

Functional divergence type I between serotypes followed a trend in agreement with the geographical pattern observed in the analysis of coevolution and functional/structural sites distribution. Functional divergence affected a significantly greater number of sites when the serotypes compared belonged to different-continent locations (*t*_19_ = 2.538, *P* = 0.02; Table 2). Moreover, widespread viruses (O, C and A) and that in the Asian continent (Asia 1) presented lower functional divergence between each other compared to the number of sites under functional divergence when serotypes confined to the African continent (SAT1, 2 and 3) were compared (*t*_7_ = 3.789, *P* = 0.007; Table 2). The distribution of sites under functional divergence also changed depending on the serotypes compared. Antigenic sites A, C and D were massively affected by functional divergence when African serotypes were compared (Figure 2). Conversely, comparison of serotypes A and O showed functional divergence in regions outside the antigenic sites, with only antigenic site A showing a moderate level of functional divergence (Figure 2). Comparison of serotypes belonging to completely different geographical areas showed functional divergence in antigenic and non-antigenic regions, probably indicating ancestral functional divergence.

### Intra-molecular co-variation generates FMDV immune-escape mutants

To determine if specific residues are important for the antigenicity of the virus through their functional/physical interaction with antigenic sites, we conducted an amino acid covariation analysis developed by Korber et al., (Korber et al. 1993). This method is implemented in the program PIMIC (Codoner, Elena and Fares, unpublished and available on request).

**Figure 3:**
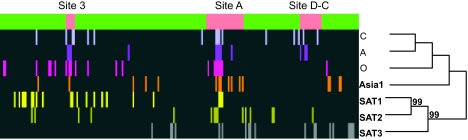
Analysis of the distribution of co-evolving sites in the different serotypes. The green horizontal bar represents the sequence of the VP1 protein of FMDV, with antigenic sites pink-labelled. Vertical bars in each horizontal lane indicate the approximate location of sites under coevolution in the serotypes compared. The different comparisons are colour-coded for clarity. The phylogenetic relationships between serotypes are also presented and internal nodes with bootstrap support values greater than 75% are indicated.

**Figure 4:**
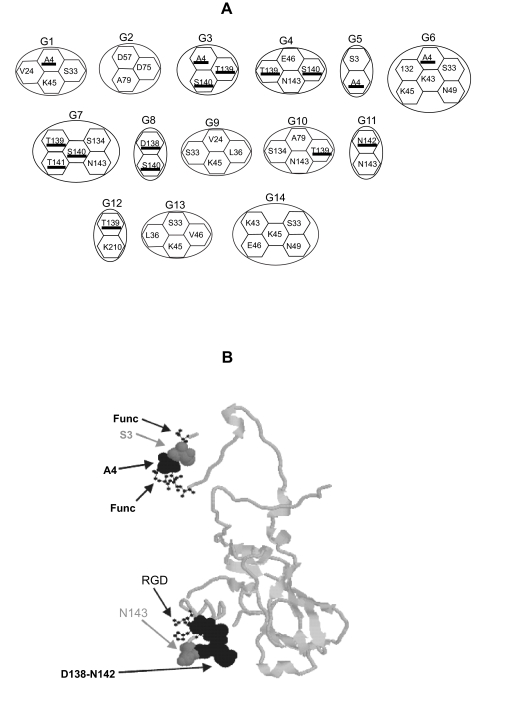
Coevolution analysis in the VP1 protein from FMDV serotype O. A) Groups of coevolution (G1 to G14) detected by the analysis. Each amino acid coevolve with all those from the same group. Underlined amino acids are those detected to have undergone adaptive evolution. B) The three-dimensional structure of the VP1 protein of FMDV (PDB entry 1QGC). Coevolving amino acid sites are highlighted in space-fill structure, whilst functional sites are defined by the ball-and-stick structure. Sites in black are those positively selected; whilst sites in grey represent those showing accelerated rates of evolution (where non-synonymous substitutions are significantly high but where non-synonymous-to-synonymous rates ratio is below 1). Functionally important sites (Func), predicted using the program CONSEQ, are marked. The RGD tri-peptide in the antigenic site A is also shown.

Sites detected as coevolving under this approach were, in all the serotypes, located in the antigenic sites A, C, D, and antigenic site 3. There was also a strong correlation between those sites co-evolving and those under positive selection. In fact, the percentage of co-evolving sites under positive selection were 100% (i.e. all sites under co-evolution were positively selected) in SAT2, 83% in A, 63% in Asia1, 33% in SAT1 and 21% in O (Figure 3). Results were not biased by the fact that positively selected sites are more prone to be detected as coevolving because in some serotypes sites under positive selection were not detected as coevolving and vice versa. Interestingly, a pattern emerges that correlates the geographic distribution of the serotype with the molecular distribution of sites under adaptive evolution or co-evolution. For example, for those serotypes widely distributed (O and A) or covering an overlapping geographical area (O, A and Asia1) several sites under adaptive evolution are found within the same group of coevolution (Figure 4A). In addition, several of the amino acid sites detected by SWAPSC to be under accelerated rates of evolution were detected as co-evolving with sites under positive selection. When adjacent sites to these accelerated amino acid residues were analysed by CONSEQ, all of them were found to be functionally important. For example, the accelerated amino acid site 3 in serotype O is coevolving with the positively selected site 4, both belonging to the coevolution group G5 (Figure 4A). This site is in physical contact (less than 4Å distant) with functionally important regions as predicted by CONSEQ (Figure 4B). The same holds for amino acid site 143 that is coevolving in several groups of co-evolution with sites under positive selection (139, 140, 141 and 142; Figure 4A). This site is contacting or is significantly close to the RGD motif (Figure 4B). Given their location, these sites are expected to be slightly deleterious and may have been thus compensated by conditional positively selected mutations as explained above. Interestingly, these sites (accelerated amino acid sites and positively selected sites) did always coincide phylogenetically further demonstrating their correlated variation. These data support thus the compensatory effect of positively selected sites in the widely distributed virus serotypes because the conditions to consider mutations as compensatory (for example, they have to coevolve with amino acid sites that have undergone accelerated rates of fixation of amino acid substitutions and these sites have to be nearby functional/structural regions) are met. Their advantageous effect however would be considered conditional to the emergence of slightly deleterious mutations and the general effect of both types of mutations is thus neutral.

**Figure 5:**
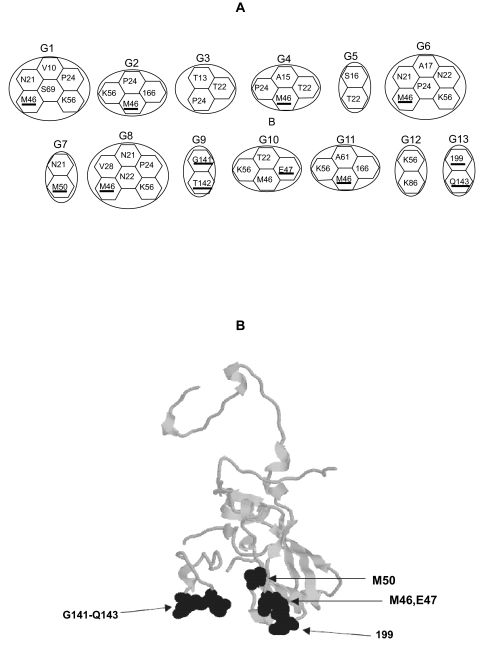
Coevolution analysis in the VP1 protein from FMDV serotype SAT1. A) Groups of coevolution (G1 to G13) detected by the analysis. All amino acids included in the same group are coevolving with each other. Underlined amino acids are those detected to have undergone adaptive evolution. B) The three-dimensional structure of the VP1 protein of foot and mouth disease virus (PDB entry 1QGC). Coevolving positively selected amino acid sites are highlighted in space-fill structure.

There was no evidence for compensatory mutations in African serotypes. In these serotypes, sites under adaptive evolution were generally included in different coevolution groups (Figure 5A). No single site under accelerated rate of evolution (with no positive selection) was found to co-evolve with spatially close positively selected sites (Figure 5B).

All four antigenic regions presented evidence of co-evolution between each other. Interestingly, the BC loop presented the highest percentage of co-evolving sites with antigenic sites A, C and D compared to other VP1 regions. A trend emerges that shows that serotype A has the greatest number of co-evolving sites in antigenic regions closely followed by C, then types O, SAT2, ASIA1, SAT1 and SAT3.

## Discussion

Previous works have attempted to answer the question of whether the appearance of a new serotype is subjected to a change in evolutionary constraints. Elena and colleagues (1992) showed that a constant rate model of evolution in the different serotypes explains the evolution of three of the FMDV serotypes. They also demonstrated that episodic positive Darwinian selection might have been operating during the evolution of CS serotype. Since this statistically elegant study was performed, several new FMDV sequences were released and statistical and computational methods developed to conduct more detailed analyses of selective constraints. In this work we have conducted a detailed and comprehensive analysis of selective constraints in the different serotypes of FMDV using all the available sequences. The importance of FMDV and the amount of data available make this virus a good system to conduct a comparative evolution analysis of virus serotypes such as the one performed here. We would however like to state that other FMDV genomic regions might clarify or modify the conclusions shown in this work. In fact, previous works have shown that the FMDV host range can be governed by non-structural proteins such as the 3A and 3B proteins (Nunez et al. 2001; Pacheco et al. 2003). Analyses of other genomic regions may provide valuable information to confirm many of the conclusions in this work.

### Heterogeneous selective constraints and the emergence of immune-escape mutants in FMDV serotypes

Adaptive evolution in FMDV has been previously detected in natural isolates and has been associated with the emergence of immune-escape and persistent mutants (Haydon et al. 2001; Tosh et al. 2003; Mittal et al. 2005). Because the method used in this study examines specific branches of the tree and regions of the alignment for adaptive evolution, additional sites to those observed previously have been detected under positive selection in FMDV type A. Splitting the evolutionary time hence in as many temporal points as branches in the FMDV tree makes the non-synonymous-to-synonymous rates ratio a more sensitive approach to detect episodic changes on selective constraints. Our results are also consistent with those of Haydon et al. (Haydon et al. 2001) as we do not find any significant level of positive selection in types C and SAT3 but do detect a considerable amount in type O. Interestingly, C and SAT 3 serotypes have almost gone to extinction to this date. As a limitation of the method used here to detect selective constraints is the fact that, even though the SWAPSC software is applicable to RNA data, selective constraints operating at synonymous sites to maintain a stable secondary RNA structure may inflate *ω* values due to the purifying selection acting on the RNA molecule. However, this would affect all the regions in the protein and thus selective constraints would not be concentrated in few biologically important protein regions.

**Figure 6:**
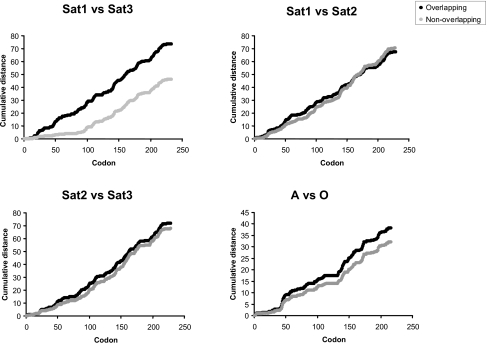
Cumulative mean Poisson-corrected pairwise distance along the sequence alignments of foot-and-mouth disease virus. Different serotypes have been compared in both overlapping geographical areas (black line) and non-overlapping areas (grey line).

On examination of the sites under adaptive evolution we find that G-H loop that ranges from amino acids 131–149 have undergone positive selection in all the serotypes. Within SAT serotypes we find Site 3 and antigenic region C under positive selection. Most of the regions under positive selection in SAT serotypes are not found in non-African serotypes, sparking speculation that there is a difference in the selective pressures governing the SAT types compared to non-African serotypes. On the other hand, the difference in the percentage of sites that have undergone positive selection is very noticeable, with SAT2 having a significantly greater percentage of sites undergoing selection compared to SAT1. This coincides with the fact that SAT2 is widely distributed in the African continent whereas SAT1 is confined to specific African regions, yet both serotypes overlap in some African territories. A plausible explanation for this correlation is that SAT2 has accumulated amino acid replacements in its VP1 protein that permitted the generation of immune-escape mutants and the emergence of variants in SAT1-SAT2 overlapping geographical areas as to minimize the competition by utilizing different niches in these areas (adaptive radiation). If this hypothesis were true, we would expect greater accelerated rates of evolution in the VP1 protein from serotypes in overlapping geographical areas compared to the same serotypes in non-overlapping areas. Examination of the Poisson-corrected amino acid distances between serotypes in overlapping regions reveals faster evolutionary rates for data sets of sequences in overlapping geographical regions for all the serotypes (Figure 6). This fact is independent of different sequence sampling dates because all the sets included sequences from similar sampling dates and were taken from the same countries. Although the analyses here can be linked to the ability of SAT1 and SAT2 to infect other hosts in addition to establish persistent infections in the buffaloes, further analyses are required to confirm the direct relationships between host range and pathogenesis and the functional divergence between both serotypes. For instance, analysing genes responsible for the host range and pathogenicity such as 3A and 3B might shed more light to this problem.

### Host-cell receptor recognition and antigenicity may be linked by positive selection and coevolution

Amino acid sites detected to be under adaptive evolution in FMDV serotypes are mostly located in antigenic regions known to interact with both, integrin receptors (Jackson et al. 1996; Jackson et al. 2000; Jackson et al. 2002) and neutralising antibodies (Verdaguer et al. 1995; Ochoa et al. 2000). It is remarkable the detection of positive selection in the RGD motif despite its direct involvement in the interaction with the host cell receptors. Experimental dispensability of the RGD tri-peptide for cell entry expands greatly the repertoire of antigenic variants of FMDV with substitutions at the antigenic site A (Martinez et al. 1997; Ruiz-Jarabo et al. 1999). FMDV exhibits an astonishing flexibility in receptor usage, as substitutions within the RGD or at neighbouring sites have been isolated in viruses from lesions in immune cattle challenged with a highly virulent FMDV (Taboga et al. 1997). The co-evolution of positively selected sites in antigenic regions, as shown in this work, also involved in the interaction with alternative cell surface receptors complements this conclusion. This co-evolution can be promoted by the position of the mobile loop ensuring interaction of residues in the G-H loop with residues located in distant regions of the VP1 protein such as the B-C loop (Verdaguer et al. 1999). We also concur with Aggarwal and Barnett (Aggarwal and Barnett, 2002) who suggested that unidentified sites outside of antigenic regions may also be important in the immune response. In this respect, our work shows non-antigenic regions under positive selection co-evolving with known antigenic sites.

### A geographical-specific pattern of adaptive coevolution in FMDV

Examination of the three-dimensional localisation of coevolving sites suggests fixation of mutations with compensatory effects in VP1 from serotypes with a wide geographical distribution. In fact, in these serotypes we identified fast-evolving sites co-evolving with positively selected sites. These accelerated sites are in contact with functionally important sites as highlighted in the three-dimensional structures. Mutations at these sites may have important negative effects on the functional regions that must be compensated by advantageous mutations at adjacent sites. Both types of mutations together would have neutral effects on the protein’s function. In addition, for those accelerated sites affecting important regions embedded in complex local structures, such as the RGD motif, several advantageous mutations at nearby sites would be needed to compensate for the deleterious effect of accelerated sites. This would explain the significant number of positively selected sites belonging to the same co-evolution group in the case of these serotypes. Conversely, those viruses with a distribution confined to the African continent showed no indication of compensatory mutations.

This pattern of coevolution may have probably been produced by fixation of slightly deleterious mutations in the widely distributed serotypes (for example, serotypes O and A) subjected to population bottlenecks due to the high host geographical mobility. Caution is however stressed in interpreting these results since high host population densities may ameliorate the effects of genetic drift caused by host mobility. Conversely, SAT serotypes are confined to specific areas of the African continent probably infecting persistently the host (for example, they have their reservoir in the African buffalo) through improving their ability to generate highly competitor immune-escape mutants. In addition, compensatory mutations and the co-evolution among antigenic sites, as demonstrated in this work, would permit fixing immune-escape mutations probably maintaining the ability to recognise host-cell receptors in these serotypes. This is in agreement with previous studies suggesting the co-evolution of antigenicity and receptor usage (Mateu et al. 1996; Domingo et al. 1999; Baranowski et al. 2001). Furthermore, the fact that mutations within the B-C loop can influence the conformational stability of the G-H loop (Parry et al. 1990) supports the importance of the co-evolving sites in both loops detected here.

### A geographical-based pattern of functional divergence between FMDV serotypes

Our analysis supports that FMDV serotypes geographically distant have diverged functionally/structurally during their independent evolution. In contrast to the African serotypes that show a geographical confinement, serotypes in the Asian continent present evidence for inter-serotype contact due to their lower functional divergence. This has been probably produced due to the greater mobility of the virus or the host that could eventually lead to bottlenecks on the viral population.

### Virus variability and survival

In this manuscript we give details about the complex dynamics of evolution of an economically important virus. Variability in RNA viruses caused by high errors rates during virus replication seems to be important for the survival of virus populations under changing environmental conditions. It has been recently shown that low fidelity increases virus pathogenesis and that selective pressures are operating at the population level (Pfeiffer and Kirkegaard 2005; Vignuzzi et al. 2006). The main conclusion of these studies is that pathogenesis is supported by Quasispecies diversity. High fixation of rates of amino acid replacements in FMDV can be hence instrumental in the survival of the viral population per se.

### Sampling bias

Sampling limitations may indeed play a factor in the interpretation of these results. In order to provide accurate reflections of the disease, there is need for complete temporal, spatial coverage and full recording of all disease events. This study attempts to maximise the geographic distribution and range of sampling times for sequences in order to obtain a random and true representation of the worldwide situation rather than single episodes or epizootics. Indeed it is inevitable that the current sequences sampled here are only a subset of those actually in nature but nevertheless they aid in our understanding of the disease. In any case, this is to our knowledge the most comprehensive study done on the evolutionary constraints on FMDV serotypes.

## Supplementary Information



## Figures and Tables

**Table 1:** Amino acid sequence regions for the VP1 protein detected as having undergone positive selection in each FMDV serotype using the program SWAPSC. Regions in bold are those detected in SAT1 and SAT2. F and S refer to functionally and structurally important sites, respectively, as predicted by a neural network analysis. Functionality refers to the antigenicity of those sites. Non-synonymous-to-synonymous rates ratio (*ω*) is shown for each region.

Serotype	Region	Functionality	Neural Network Prediction	*ω*
**SAT-2**	18–40	Site 3	F, S	3.869
	**43–62**	BC loop/Site 3	F, S	4.117
	65–68		F, S	2.207
	74–77		F	8.754
	81–87		F	3.525
	**97–101**		F	4.058
	108–114		F, S	2.903
	117–120		F, S	1.813
	123–128		F, S	4.015
	134–143	Site A	F, S	3.00
	151–162	G-H loop	F, S	4.634
	172–178		F, S	2.886
	189–192	Site D/C	F, S	8.111
	**196–203**	C-T loop/Site C	F	6.399
	**213–218**		F	5.067
**SAT-1**	**45–50**	B-C loop	F	1.526
	**95–101**		F	1.658
	136–149	G-H loop	F	7.080
	**199–211**	Site D/C	F	7.662
	**216–244**		F	5.313
**ASIA 1**	92–105		F	6.540
	135–143	G-H loop	F	4.982
	148–160	Site A	F, S	3.356
	209–214		F	8.965
**A**	44–51	B-C loop/Site 3	S	2.030
	131–148	G-H loop/	F	1.415
	164–217	H-I loop/Site D/Site C	F, S	2.586
**O**	4–6		F	5.311
	68–70		F	2.500
	81–83		F	2.167
	85–87		S	3.107
	96–98		F	2.500
	137–142	G-H loop		3.371
	144–153	G-H loop	F	3.216
	177–179		F, S	2.682
	197–199	C-T loop/Site C		8.131
	201–204	C-T loop/Site C	F	2.167
	211–231		F	4.782

**Table 2:** Hemi-matrix of the number of sites under functional divergence type I among FMDV serotypes.

Serotype	SAT1	SAT2	SAT3	ASIA1	C	A	O
SAT1	-						
SAT2	11	-					
SAT3	14	11	-				
ASIA1	13	22	18	-			
C	6	21	30	7	-		
A	15	33	80	7	5	-	
O	15	14	22	6	0	7	-
