# Supplementary material for: Unravelling Selection Shifts Among Foot-and-Mouth Disease Virus (FMDV) Serotypes
Source: Evol Bioinform Online. 2007 Feb 11;2:211–25. (PMC2674665)
Supplement: Supplementary file 1 [file EBO-2-Fares.pdf]

SUPPLEMENTARY INFORMATION

Table 1. Sampling dates and geographic ranges for FMDV serotypes.

Serotype	N[a]	Date range	Geographic Sampling (N)	π[b]
A	182	1932-2001	Argentina (32), Brazil (7), Cameroon (38), Colombia (2), France (1), Germany (5), Holland (1), India (75), Iraq (3), Iran (1), Italy (1), Kenya (2), Peru (1), Philippines (1), Spain (3), Thailand (1), Turkey (2), Uruguay (2), UK (1), USSR (1), Venezuela (2)	0.140
O	203	1947-2001	Angola (2), Argentina (14), Armenia (1), Bahrain (2), Bangladesh (1), Belgium (1), Bhutan (1), Brazil (2), Burundi (2), Burkina Faso (3), Cameroon (12), China (8), Cote de Ivory (1), Eritrea (2), Ethiopia (17), Ghana (4), Hong Kong (15), India (1), Indonesia (4), Iran (5), Iraq (2), Israel (1), Italy (1), Japan (1), Kenya (4), Kuwait (1), Laos (1), Lebanon (1), Malaysia (1), Mongolia (1), Myanmar (1), Niger (3), Pakistan (1), Philippines (4), Poland (1), Russia (12), Saudi Arabia (2), South Africa (6), South Korea (4), Sri Lanka (1), Taiwan (29), Tanzania (1), Thailand (1), Turkey (3), Unknown (1), Uruguay (1), Uganda (1), UAE (1), UK (12), Venezuela (1), Vietnam (4)	0.104
C	30	1953-1994	Argentina (13), Belgium (1), Brazil (2), Denmark (1), France (2), Germany (1), Italy (1), Spain (3), Switzerland (2), UK (2), Unknown (2)	0.080

Asia 1	70	1954-1999	India (66), Lebanon (1), Israel (2), Pakistan (1)	0.085
SAT 3	48	1960-1999	Botswana (4), Kenya (1), Namibia (5), South Africa (13), Uganda (1), Unknown (1), Zimbabwe (18), Zambia (5)	0.168
SAT 1	68	1962-2000	Botswana (9), Israel (1), Namibia (4), Nigeria (19) Niger (4), South Africa (8), Swaziland (2) Tanzania (2), Unknown (3), UK (1), Zimbabwe (12), Zambia (3)	0.203
SAT 2	64	1948-2001	Angola (1), Botswana (4), Burundi (1), Eritrea (1), Gambia (2), Ghana (2), Kenya (4), Malawi (1), Mozambique (2), Namibia (2), Nigeria (1), Rwanda (1), Saudi Arabia (1), Senegal (4), South Africa (25), Unknown (1), Zaire (1), Zambia (6), Zimbabwe (4)	0.157

[a] Number of sequences used in the study

[b] Mean pairwise DNA genetic distance

Table 2. FMDV serotypes, isolate names and GenBank accession numbers.

Serotype	Isolate name	GeneBank Accession Number
A	25DEMAYO/ARG/87	AJ306220
A	ARGP64/01	AY593785
A	ALEM/ARG/81	AJ306219
A	ARGP55/01	AY593784
A	ARGENTINA/79	K03345
A	ARGENTINA/68	AJ308694
A	A25/ARGENTINA/59	AY593769
A	ARGENTINA/76	AJ409219
A	A26/ARGENTINA/66	AY593770
A	A17/AGUARULBOS/67	AY593757
A	A4/SPAIN	AY593778
A	A24/ARGENTINA/65	AY593767
A	A5/ALLIER/60	AY593780
A	AYACUCHO/ARG/90	AJ308696
A	ARGENTINA/ARECO/01	AY593783
A	ARGENTINA/00	AY593782
A	BAGE/77	AY593787
A	A16/BELEM/59	AY593756
A	BRAZIL/79	AY593788
A	BAHIABLANCA/ARG/71	AJ308695
A	BAYERN/BAVERIA/71	AY593759
A	BRAZIL/70	AY593753
A	COLOMBIA/67	AY593771
A	CASTELLANOS/ARG/87AF2	U62260
A	CAR/1/76	AY254443
A	CASTELLANOS/ARG/87AFC2	U62257
A	CASTELLANOS/ARG/87	AJ306222
A	CASTELLANOS/ARG/87AFC3	U62258
A	CAR/5/85	AY254442
A	CASTELLANOS/ARG/87AFC1	U62256
A	CASTELLANOS/ARG/87CLONEA	U62255
A	CASTELLANOS/ARG/87AF1	U62259
A	CANEFA/61	AY593789
A	CAR/15/2000	AY254413
A	CAR/12/2000	AY254410
A	A24/CRUZEIRO/55	AY593768
A	CAR/14/75	AY254440

A	CORDOBA/ARG/90	AJ308697
A	CAR/13/2000	AY254412
A	CORDOBA/ARG/92	AJ308700
A	CUNDINAMARCA/COL/76	K03341
A	CAR/14/2000	AY254411
A	CAR/4/86	AY254441
A	CORRIENTES/ARG/92	AJ308701
A	DN1/100/08	AY254431
A	DNL/100/05	AY254418
A	DNL/085/01	AY254423
A	DNL/085/08	AY254424
A	DNL/085/09	AY254425
A	DN1/085/10	AY254432
A	DMA/112/03	AY254406
A	DNL/100/06	AY254416
A	DAM/112/06	AY254407
A	DMA/112/01	AY254405
A	FKE/066/08	AY254437
A	GENERALLOPEZ/01	AY593790
A	HOLLAND/42	AY593751
A	IND/233/99	AF390621
A	IND/252/99	AF390626
A	IND/68/2001	AF390659
A	IND/258/99	AF390630
A	IND/76/96	AF390662
A	IND/237/99	AF390623
A	IND/490/97	AF390652
A	IND/170/88	AF390612
A	IND/160/90	AF390608
A	IND/135/99	AF390603
A	IND/61/88	AF390657
A	IND/110/99	AF390593
A	IND/302/88	AF390641
A	IND/139/99	AF390605
A	IND/395/88	AF390645
A	IND/253/98	AF390627
A	IND/455/98	AF390650
A	IND/2/93	AF390635
A	IRAQ/24/64	AY593762
A	IRAQ/26/64	AY593763

A	IND/299/99	AF390640
A	IRAQ/70	AY593764
A	IND/236/99	AF390622
A	IRAN/98	AY593791
A	IND/257/96	AF390629
A	IND/271/97	AF390632
A	IND/294/99	AF390636
A	IND/163/97	AF390611
A	IND/55/86	AF390655
A	IND/156/97	AF390607
A	IND/161/97	AF390609
A	IND/256/98	AF390628
A	IND/456/98	AF390651
A	IND/270/96	AF390631
A	IND/172/94	AF390615
A	IND/96/96	AF390673
A	IND/287/96	AF390633
A	IND/83/97	AF390667
A	IND/289/96	AF390634
A	IND/84/97	AF390669
A	IND/93/96	AF390672
A	IND/299/94	AF390639
A	IND/138/99	AF390604
A	IND/148/93	AF390606
A	IND/92/96	AF390671
A	IND/24/2001	AF390624
A	IND/172/2000	AF390614
A	IND/80/2000	AF390665
A	IND/78/2000	AF390664
A	IND/84/2000	AF390668
A	IND/38/2000	AF390643
A	IND/81/2000	AF390666
A	IND/40/2000	AF390646
A	IND/126/2000	AF390599
A	IND/42/2000	AF390648
A	IND/104/2000	AF390592
A	IND/128/2000	AF390600
A	IND/408/97	AF390647
A	IND/432/97	AF390649
A	IND/297/99	AF390637

A	IND/173/2000	AF390616
A	IND/21/90	AF390620
A	IND/39/2000	AF390644
A	IND/248/96	AF390625
A	IND/174/2000	AF390617
A	IND/115/2000	AF390595
A	IND/16/2000	AF390610
A	IND/53/2000	AF390654
A	IND/134/2000	AF390602
A	IND/50/2000	AF390653
A	IND/13/2000	AF390601
A	IND/298/99	AF390638
A	IND/10/2000	AF390591
A	IND/119/2000	AF390597
A	IND/11/2000	AF390594
A	IND/123/99	AF390598
A	IND/19/2000	AF390618
A	IND/67/2000	AF390658
A	IND/116/2000	AF390596
A	KENYA/6/65	AY593766
A	KENYA/64	AY593761
A	MBA/094/09	AY254435
A	MBD/096/03	AY254434
A	MKI/099/01	AY254409
A	MFA/055/03	AY254408
A	MECKLENBURG/68	AY593776
A	MFA/057/01	AY254419
A	PEHUAJO/ARG/92	AJ308702
A	PERU/69	AY593773
A	PHILLIPINES/75	AY593793
A	PARMA/62	AY593792
A	RIVADAVIA/ARG/91	AJ308699
A	SABANA/85	AY593794
A	SPAIN/69	AY593774
A	SANIGNACIO/ARG/90	AJ308698
A	SPAIN/59	AY593754
A	TURKEY/65	AY593765
A	TURKEY/72	AY593772
A	THAILAND/60	AY593755
A	TRENQUELAUQUEN/01	AY593786

A	USSR/1/64	AY593760
A	URUGUAY/98	AY593802
A	URUGUARY/01	AY593801
A	UTRACAN/ARG/87	AJ306221
A	VLV/122/04	AF254420
A	VLV/122/06	AY254421
A	VLV/122/20	AY254422
A	VLV/122/02	AY254426
A	VDI/044/66	AY254439
A	VLV/122/16	AY254417
A	VLV/122/13	AY254428
A	VLV/122/03	AY254429
A	VLV/122/08	AY254430
A	VBM/153/09	AY254433
A	VALLESTRAIN 119	AY593752
A	VNE/126/09	AY254415
A	VLV/122/19	AY254414
A	VLV/122/11	AY254427
A	VNE/126/01	AY254436
A	VENEZUELA/70	AY593775
A	VENCESLAU/76	AY593803
A	WESTERWALD/51	AY593781
A	WG/72	AY593779
A	WG/42	AY593777
A	ZULIA/67	AY593758
C	ARGENTINA/69	AY593809
C	ARGENTINE/69	M90377
C	ARGENTINA/83	M90371
C	ARGENTINA/84	M90377
C	BRESCIA/ITALY/64	M86532
C	C-S18	M18899
C	C-S9	M18898
C	GRALROCA/ARG/03/93	AJ306215
C	GRAL/LAMADRID/ARG/93	AJ306213
C	GRALROCA/ARG/02/93	AJ306214
C	GRALVILLEGAS/ARG/93	AJ306217
C	HAUTELOIRE/FRANCE/69	M84360
C	INDAIA/71	AY593806
C	LOUPOIGNE/BELGIUM/53	M90369
C	NOVILLE/SWITZERLAND/65	M90379

C	NOVILLE/65	AY593804
C	OBERBAYERN/60	AY593805
C	RIVADAVIA/ARG/93	AJ292208
C	RIART/SPAIN/82	M18900
C	RESENDE/55	L08386
C	SANCRISTOBAL/ARG/94	AJ306218
C	SANANTONIODEGILES/ARG/92	AJ308704
C	SALTO/ARG/91	AJ308703
C	TURUP/DENMARK/61	M90370
C	TIERRADELFUEGO/66	AY593808
C	UNKNOWNSTRAIN1	M63637
C	UNKNOWNSTRAIN2	M63638
C	VOSGES/FRANCE/60	M90375
C	WALDMAN/149	AY593810
C	997/UK/53	M90380
SAT1	BEC/1/70	AY593838
SAT1	BOT/37/98	AF137405
SAT1	BOT/25/98	AF137404
SAT1	BOT/17/77	AF137401
SAT1	BOT/24/77	AF301423
SAT1	BOT/2/98	AF137402
SAT1	BOT/14/98	AF137403
SAT1	BOT/1/68	AY593845
SAT1	BOT/8/98	AF301432
SAT1	ISRL/4/62	AY593844
SAT1	KNP/17/96	AF301431
SAT1	KNP/75/98	AF137406
SAT1	NIG/20/75	AF431713
SAT1	NIG/24/75	AF431714
SAT1	NAM/306/98	AF137408
SAT1	NAM/308/98	AF137410
SAT1	NIG/25/75	AF431715
SAT1	NIG/2/79	AF431728
SAT1	NIG/14/76	AF431725
SAT1	NIG/14/75	AF431709
SAT1	NIG/5/81	AF431730
SAT1	NIG/15/75	AF431710
SAT1	NIG/10/81	AF431731
SAT1	NIG/2/76	AF431722
SAT1	NIG/3/80	AF431729

SAT1	NIG/6/76	AF431716
SAT1	NIG/1/76	AF431721
SAT1	NGR/2/76	AF431718
SAT1	NGR/1/76	AF431717
SAT1	NIG/7/76	AF431726
SAT1	NIG/20/76	AF431727
SAT1	NAM/272/98	AF137407
SAT1	NGR/5/76	AF431720
SAT1	NAM/288/98	AF301433
SAT1	NGR/4/76	AF431719
SAT1	NIG/8/76	AF431724
SAT1	NIG/11/75	AF431711
SAT1	NIG/5/76	AF431723
SAT1	NIG/17/75	AF431712
SAT1	RHOD/5/66	AY593846
SAT1	RV/11/37	AY593839
SAT1	SWL/1/00	AF446016
SAT1	SAR/34/00	AF446015
SAT1	SA/61	AY593842
SAT1	SAR/8/00	AF446014
SAT1	SWL/4/00	AF446017
SAT1	SWA/1/49	AY593840
SAT1	SWA/40/61	AY593843
SAT1	SAR/1/00	AF446010
SAT1	SAR/2/00	AF446011
SAT1	SAR/4/00	AF446013
SAT1	SAR/3/00	AF446012
SAT1	SR/2/58	AY593841
SAT1	TAN/1/99	AF301434
SAT1	TAN/2/99	AF301435
SAT1	ZIM/14/98	AF137411
SAT1	ZIM/6/99	AF301438
SAT1	ZIM/3/96	AF283455
SAT1	ZIM/7/99	AF301439
SAT1	ZIM/25/90	AF301426
SAT1	ZIM/GN34/91	AF301429
SAT1	ZIM/47/90	AF301428
SAT1	ZIM/HV11/90	AF301424
SAT1	ZIM/26/90	AF301427
SAT1	ZIM/14/90	AF301425

SAT1	ZIM/3/95	AF301430
SAT1	ZAM/1/99	AF301436
SAT1	ZIM/5/99	AF301437
SAT2	ANG/4/74	AF479417
SAT2	BOT/1/98	AF367122
SAT2	BOT/18/98	AF367123
SAT2	BOT/31/98	AF367125
SAT2	BUN/1/91	AF367111
SAT2	BOT/29/98	AF367124
SAT2	ERI/12/98	AF367126
SAT2	GAM/8/79	AF479410
SAT2	GHA/2/90	AF479415
SAT2	GHA/8/91	AF479416
SAT2	GAM/9/79	AF479411
SAT2	KNP/19/88	AF367106
SAT2	KEN/9/99	AF367133
SAT2	KNP/20/88	AF367107
SAT2	KEN/7/99	AF367132
SAT2	KEN/5/99	AF367131
SAT2	KEN/11/60	AY593849
SAT2	KNP/18/88	AF367138
SAT2	KNP/16/88	AF367104
SAT2	KNP/1/92	AF367114
SAT2	KNP/32/92	AF367115
SAT2	KNP/18/95	AF367118
SAT2	KNP/31/95	AF367119
SAT2	KNP/7/88	AF367103
SAT2	KNP/183/91	AF367112
SAT2	KNP/19/89	AF367110
SAT2	KNP/17/88	AF367105
SAT2	KNP/2/89	AF367109
SAT2	MOZ/1/79	AF367137
SAT2	MOZ/4/83	AF367101
SAT2	MAL/3/75	AF367099
SAT2	NIG/2/75	AF367139
SAT2	NAM/292/98	AF367128
SAT2	NAM/304/98	AF367129
SAT2	PAL/5/83	AF367102
SAT2	RWA/1/00	AF367134
SAT2	RHOD/48	AY593847

SAT2	SAR/5/01	AY442907
SAT2	SAR/9/01	AY442911
SAT2	SAR/3/01	AY442905
SAT2	SAU/6/00	AF367135
SAT2	SAR/2/01	AY442904
SAT2	SEN/5/75	AF367140
SAT2	SEN/3/83	AF479413
SAT2	SAR/8/01	AY442910
SAT2	SEN/7/79	AF479412
SAT2	SAR/11/01	AY442913
SAT2	SEN/7/83	AF479414
SAT2	SAR/6/01	AY442908
SAT2	SAR/4/01	AY442906
SAT2	SAR/1/01	AY442903
SAT2	SAR/10/01	AY442912
SAT2	SAR/7/01	AY442909
SAT2	NAM/286/98	AF367127
SAT2	ZIM/267/98	AF367130
SAT2	ZIM/1/00	AF367136
SAT2	ZAM/9/93	AF367116
SAT2	ZAM/10/93	AF367117
SAT2	ZAM/7/96	AF367120
SAT2	ZAM/10/96	AF367121
SAT2	ZIM/GN10/91	AF367113
SAT2	ZAI/1/82	AF367100
SAT2	ZIM/1/88	AF367108
SAT2	106/67	AY593848
SAT3	BOT/6/98	AY258050
SAT3	BOT/9/98	AY168816
SAT3	BEC/1/65	AY593853
SAT3	BEC/20/61	AY593851
SAT3	KNP/5/96	AY168810
SAT3	KNP/6/88	AY168791
SAT3	KNP/3/96	AY168809
SAT3	KNP/14/96	AY168813
SAT3	KNP/3/94	AY168802
SAT3	KNP/15/96	AY168814
SAT3	KNP/33/94	AY168803
SAT3	KNP/36/93	AY258048
SAT3	KNP/11/96	AY168812

SAT3	KNP/9/96	AY168811
SAT3	KNP/44/94	AY168804
SAT3	KENYA/11/60	AY593852
SAT3	NAM/294/98	AY258052
SAT3	NAM/5/94	AY168806
SAT3	NAM/274/98	AY168817
SAT3	NAM/1/94	AY168805
SAT3	NAM/287/98	AY258051
SAT3	RHO/3/78	AY168790
SAT3	SA/57/59	AY593850
SAT3	SAR/1/80	AY258042
SAT3	SAR/336/98	AY168818
SAT3	UGA/2/97	AY192556
SAT3	ZIM/32/90	AY258043
SAT3	ZIM/3/99	AY168820
SAT3	ZIM/3/94	AY168807
SAT3	ZIM/1/91	AY168798
SAT3	ZAM/5/93	AY168800
SAT3	ZIM/4/99	AY168821
SAT3	ZIM/5/91	AY168799
SAT3	ZIM/11/94	AY168808
SAT3	ZIM/13/91	AY248047
SAT3	ZIM/GN9/91	AY168796
SAT3	ZIM/31/90	AY168793
SAT3	ZIM/HV5/90	AY168792
SAT3	ZIM/11/91	AY258046
SAT3	ZIM/CK7/91	AY258045
SAT3	ZAM/7/93	AY168801
SAT3	ZIM/CK4/91	AY168794
SAT3	ZAM/1/93	AY258049
SAT3	ZIM/CK5/91	AY258044
SAT3	ZAM/11/96	AY168815
SAT3	ZIM/CK10/91	AY168795
SAT3	ZIM/1/99	AY168819
SAT3	ZIM/GN39/91	AY168797
Asia1	IND/108/99	AF392902
Asia1	IND/92/99	AF392925
Asia1	IND/126/99	AF392903
Asia1	IND/69/99	AF392947
Asia1	IND/277/99	AF392909

Asia1	IND/286/99	AF392911
Asia1	IND/324/98	AF392938
Asia1	IND/397/97	AF392942
Asia1	IND/102/99	AF392897
Asia1	IND/388/97	AF392915
Asia1	IND/104/99	AF392898
Asia1	IND/386/97	AF392914
Asia1	IND/105/99	AF392899
Asia1	IND/390/97	AF392940
Asia1	IND/127/99	AF392929
Asia1	IND/15/95	AF390680
Asia1	IND/339/96	AF392939
Asia1	IND/82/96	AF390705
Asia1	IND/125/98	AF392928
Asia1	IND/57/95	AF390696
Asia1	IND/51/93	AF392923
Asia1	IND/70/96	AF390698
Asia1	IND/314/94	AF392937
Asia1	IND/73/96	AF390701
Asia1	IND/43/95	AF392916
Asia1	IND/50/95	AF390694
Asia1	IND/278/99	AF392934
Asia1	IND/80/96	AF390703
Asia1	IND/256/97	AF392906
Asia1	IND/89/96	AF390706
Asia1	IND/177/88	AF392904
Asia1	IND/81/96	AF390704
Asia1	IND/2/90	AF392912
Asia1	IND/26/95	AF390687
Asia1	ISRL/3/63	AY593796
Asia1	IND/175/94	AF392931
Asia1	IND/267/88	AF390686
Asia1	IND/173/96	AF390681
Asia1	IND/152/94	AF392930
Asia1	IND/10/91	AF390674
Asia1	IND/305/94	AF392936
Asia1	IND/19/89	AF390684
Asia1	IND/24/95	AF392933
Asia1	IND/120/91	AF392927
Asia1	IND/22/88	AF390685

Asia1	IND/45/89	AF390691
Asia1	IND/33/96	AF390689
Asia1	IND/17/91	AF390682
Asia1	IND/43/96	AF390690
Asia1	IND/13/91	AF390677
Asia1	IND/44/98	AF392943
Asia1	IND/53/93	AF390695
Asia1	IND/63/72	Y09949
Asia1	IND/47/95	AF392921
Asia1	IND/132/90	AF390676
Asia1	IND/396/97	AF392941
Asia1	IND/46/87	AF390692
Asia1	IND/247/92	AF392932
Asia1	IND/120/88	AF390675
Asia1	IND/116/90	AF392926
Asia1	IND/10/86	AF392901
Asia1	IND/491/97	AF392922
Asia1	IND/68/92	AF392946
Asia1	IND/49/93	AF392945
Asia1	IND/82/86	AF392924
Asia1	IND/75/86	AF390702
Asia1	IND/107/99	AF392900
Asia1	KFAR/KELA/LEBANON/83	AJ294931
Asia1	KIMRON/63	AY593797
Asia1	PAK/54	AY593795
O	ARGENTINA/65	AY593814
O	ARG/77	AJ308705
O	AYACUCHO/ARG/90	AJ308707
O	ANG/1/75	AF300811
O	ANG/10/74	AF300810
O	A/58	AJ131469
O	BKF/2/92	AF274296
O	BKF/1/92	AF300804
O	BKF/3/92	AF300805
O	BFS46/67	AY593816
O	BRESCIA/47	AY593826
O	BAR/8/98	AJ318825
O	BHU/1/98	AJ318826
O	BUR/2/89	AJ318824
O	BUR/6/89	AJ294905

O	BAR/2/97	AJ318824
O	BFS18/67	AY593815
O	BRUGGE/73	AY593817
O	BAN/3/96	AJ303483
O	CHA/4/99	AJ318833
O	CHUNHWA/188	AF095883
O	CAM/12/94	AJ294907
O	CHA/3/99	AJ318832
O	CHUNHWA/158	AF095878
O	CAMPOS/58	AY593818
O	CAM/11/94	AJ294906
O	CHA/2/99	AJ318831
O	CHAPALEUFU/ARG/92	AJ308708
O	CAM/2/98	AJ294909
O	CAM/1/98	AJ294908
O	CAM/3/98	AJ294910
O	CAM/6/99	AJ318827
O	CASEROS/67	AY593821
O	CIV/8/99	AJ303485
O	CAR/12/88	AY254400
O	CAR/17/00	AY254403
O	CAM/2/2000	AJ318828
O	CANEFA/64	AY593820
O	CHA/1/99	AJ318830
O	CAMPOS/94	AY593819
O	CAM/4/2000	AJ318829
O	ETH/16/2001	AY283394
O	ETH/3/96	AY283392
O	ETH/19/83	AY283378
O	ETH/8/94	AY283384
O	ETH/1/79	AY283376
O	ETH/5/95	AY283388
O	ETH/1/95	AY283387
O	ETH/3/79	AY283377
O	ETH/12/90	AY283381
O	ETH/9/92	AY283382
O	ETH/3/90	AY283379
O	ETH/24/94	AY283385
O	ETH/15/2001	AY283393
O	ETH/30/94	AY28338

O	ETH/2/93	AY283383
O	ERI/2/96	AY283391
O	ERI/1/96	AY283390
O	ETH/8/90	AY283380
O	ETH/22/2001	AY283395
O	GHA/6/93	AF300807
O	GHA/9/93	AF300809
O	GRALCONESA/ARG/94	AJ292206
O	GHA/5/93	AF300806
O	GD/CHINA/86	AJ131468
O	GHA/7/93	AF300808
O	HKN/1/73	AJ294912
O	HK/93	AJ131470
O	HSINCHU/0/79	AF095867
O	HKN/19/73	AJ294913
O	HKN/3/75	AJ294915
O	HKN/33/77	AJ294916
O	HSINCHU/128	AF095873
O	HKN/14/82	AJ294917
O	HKN/6/83	AJ294919
O	HKN/7/85	AJ294920
O	HKN/17/82	AJ294918
O	HKN/1/99	AJ294925
O	HKN/10/99	AJ318836
O	HKN/7/96	AJ294922
O	HKN/20/96	AJ294924
O	HSINCHU/189	AF095884
O	HKN/12/91	AJ294921
O	HKN/16/96	AJ294923
O	IRN/24/99	AJ318839
O	IRN/9/99	AJ318838
O	INDIA/62	AY593828
O	IRN/15/97	AJ318837
O	IRAN/66	AY593834
O	ISA/9/74	AJ303502
O	IRQ/30/2000	AJ303499
O	ISR/3/99	AJ318842
O	ISA/1/62	AJ303500
O	IRN/16/2000	AJ318840
O	ISA/1/74	AJ303501

O	IRQ/26/2000	AJ318841
O	JAV/5/72	AJ303509
O	JPN/2000	AB050978
O	KAOHSIUNG/190	AF095885
O	KEN/2/95	AJ303514
O	KEN/77/78	AF300812
O	KAOHSIUNG/153	AF095877
O	KUW/4/97	AJ318843
O	KEN/83/79	AJ303511
O	KEN/10/95	AY283389
O	LUJAN/ARG/83	AJ308706
O	LEB/1/98	AJ318845
O	LAO/2/2000	AJ318844
O	MOG/2000	AJ318847
O	MYA/1/98	AJ303521
O	MAY/2/2000	AJ318846
O	MANISA/69	AY593823
O	M11	AY593822
O	MIAOLI/165	AF095879
O	NGR/1/88	AF300801
O	NGR/2/88	AF300802
O	NGR/3/88	AF300803
O	N1685/RUS/95	AJ004680
O	N194	AJ004677
O	NANTOW/089	AF095868
O	N1492	AJ004679
O	N1451	AJ004669
O	N738	AJ004663
O	N1491	AJ004672
O	N850	AJ004665
O	N1618	AJ004678
O	N1427	AJ004668
O	N1467	AJ004670
O	N822	AJ004664
O	O/SKR/2000	AF377945
O	POLAND/59	AY593830
O	GRAL/PUEYRREDON/ARG/93	AJ292209
O	PAK/1/97	AJ303526
O	PIRBRIGHT/65	AY593829
O	PHI/7/96	AJ294926

O	PHI/5/99	AJ318849
O	PINGTUNG/060	AF095865
O	PENGHU/99	AY593833
O	PHILIPPINES/2/58	AY593812
O	PHILIPPINES/58	AY593811
O	RIVADAVIA/ARG/94	AJ306212
O	RIVADAVIA/ARG/93	AJ292208
O	SKR/1/2000	AJ318854
O	SAR/12/00	AY009087
O	SAR/19/2000	AJ539140
O	SAR/13/00	AY009088
O	SAU/38/98	AJ318852
O	SAR/11/00	AF306646
O	SAR/15/00	AF306647
O	SKR/2000	AF428246
O	SAU/2/97	AJ318851
O	SRL/2/97	AJ303531
O	SKR/00	AY593824
O	SAR/1/2000	AJ318860
O	TAIPEI/110	AF095870
O	TAOYUAN/113	AF095872
O	TAOYUAN/018	AF095863
O	TAICHUNG/186	AF095882
O	TAICHUNG/0/77	AF095866
O	TAW/83/97	AJ296322
O	TAITUNG/111	AF095871
O	TAW/2/99	AJ294927
O	TAIPEI/109	AF095869
O	TIBET/CHA/99	AJ539138
O	TAN/7/98	AJ296320
O	TUR/6/98	AJ318855
O	TAINAN/041	AF095864
O	TAW/81/97	AJ296321
O	TAINAN/168	AF095880
O	TUR/1/96	AJ296325
O	TAINAN/181	AF095881
O	TAI/4/99	AJ303536
O	TAIPEI/150	AF095876
O	TAIPEI/145	AF095875
O	TAIWAN/97	AY593835

O	TAW/4/99	AJ294928
O	UKG/11/2001	AJ311723
O	UKG/10/2001	AJ311722
O	UKG/6/2001	AJ311721
O	UK/2001	AY593836
O	URUGUAY/63	AY593837
O	UK/FB/2001	AY593832
O	UK/ED/2001	AY593831
O	UKG/35/2001	AJ539141
O	UGA/5/96	AJ296327
O	UAE/7/97	AJ318856
O	UKG/12/2001	AJ311724
O	UKG/3/2000	AJ311720
O	VILLARINO/ARG/03/93	AJ292207
O	VDI/044/70	AY254402
O	VDI/044/62	AY254401
O	VILLARINO/ARG/01/93	AJ292205
O	VALLEE/39	AY593825
O	VIT/2/97	AJ294929
O	VIT/17/99	AJ318858
O	VIT/3/97	AJ294930
O	VIT/7/97	AJ296328
O	VENEZUELA/71	AY593827
O	XJ1	AY373583
O	YUNLIN/136	AF095874
O	1691/ARM/96	AJ318823
O	1696/GRG/97	AJ318834
O	1734/RUS/2000	AJ318850
